# PD-YOLO: a novel weed detection method based on multi-scale feature fusion

**DOI:** 10.3389/fpls.2025.1506524

**Published:** 2025-04-08

**Authors:** Shengzhou Li, Zihan Chen, Jialong Xie, Hewei Zhang, Jianwen Guo

**Affiliations:** School of Mechanical Engineering, Dongguan University of Technology, Dongguan, China

**Keywords:** weed detection, object detection, YOLO, multi-scale feature fusion, dynamic detection head

## Abstract

**Introduction:**

The deployment of robots for automated weeding holds significant promise in promoting sustainable agriculture and reducing labor requirements, with vision based detection being crucial for accurate weed identification. However, weed detection through computer vision presents various challenges, such as morphological similarities between weeds and crops, large-scale variations, occlusions, and the small size of the target objects.

**Methods:**

To overcome these challenges, this paper proposes a novel object detection model, PD-YOLO, based on multi-scale feature fusion. Building on the YOLOv8n framework, the model introduces a Parallel Focusing Feature Pyramid (PF-FPN), which incorporates two key components: the Feature Filtering and Aggregation Module (FFAM) and the Hierarchical Adaptive Recalibration Fusion Module (HARFM). These modules facilitate efficient feature fusion both laterally and radially across the network. Furthermore, the inclusion of a dynamic detection head (Dyhead) significantly enhances the model’s capacity to detect and locate weeds in complex environments.

**Results and discussion:**

Experimental results on two public weed datasets demonstrate the superior performance of PD-YOLO over state-the-art models, with a modest increase in computational cost. PD-YOLO improves the mean average precision (mAP) by 1.7% and 1.8% on the CottonWeedDet12 dataset at thresholds of 0.5 and 0.5-0.95, respectively. This research not only presents an efficient and accurate weed detection method but also offers new insights and technological advances for automated weed detection in agriculture.

## 1 Introduction

Weeds are one of the major factors affecting agriculture. Currently, the damage caused by weeds to agriculture reaches as high as 34% (Oerke, 2006). For decades, herbicides have been widely adopted as the preferred method for weed management in global agriculture; however, herbicides often have adverse environmental side effects (Kudsk and Streibig, 2003). With the development of precision agriculture and the widespread use of intelligent agricultural machinery, robots for weed control hold great potential in building environmentally friendly agriculture and reducing labor demands (You et al., 2020). Intelligent robots rely on real-time weed detection systems with high accuracy to locate weeds, but weed detection in actual farmland environments still faces the following challenges.

Firstly, the coexistence of inter-class phenotypic similarity and intra-class morphological variation poses significant challenges. Weeds and crops exhibit substantial overlap in color features, particularly during the seedling stage, with notable similarities in leaf shape and texture characteristics during growth (Hasan et al., 2021). For instance, Italian ryegrass (Lolium multiflorum) and wheat are visually indistinguishable without expert knowledge (Bansal et al., 2024), rendering traditional detection methods ineffective. Compounding this complexity, dynamic variations in leaf color, morphology, and texture occur across different growth stages of the same weed species (Veeragandham and Santhi, 2021), hindering the establishment of stable feature representations in detection models. Studies have shown that YOLOX and YOLOv8 models experienced accuracy declines of 14.5% and 14.2%, respectively, in identifying eight cross-season weed categories common in cotton fields. This degradation primarily stems from seasonal variations in lighting, background conditions, and weed growth states (Deng et al., 2024).

Secondly, the sheer diversity of weed species (Chen et al., 2024), combined with the simultaneous presence of weeds at varying developmental stages within agroecosystems, creates scale differences spanning three orders of magnitude. Empirical analysis using the Weed25 dataset revealed significant disparities in recognition performance: Asiatic smartweed (Polygonum aviculare) achieved a mean average precision (mAP) of 62.92%, while velvetleaf (Abutilon theophrasti) reached 99.70% (Wang et al., 2022). Such scale heterogeneity complicates the development of algorithms capable of effectively detecting multi-category weeds. Furthermore, leaf overlap and weed occlusion in dense scenarios exacerbate differentiation and detection challenges (Wang et al., 2019). In cabbage fields, weed detection not only struggles with color similarity but also contends with illumination variations and leaf occlusion, leading to suboptimal performance in direct detection methods addressing these issues (Sun et al., 2024).

Finally, the high-density distribution and small size of weed targets frequently result in missed detections and false positives. Wu et al. (2023). proposed an enhanced YOLO-V4 model tailored for small weed detection in farmland, improving the mAP by 4.2%. However, despite advancements in lightweight performance, the parameter count of model remains high at 42.54 million, posing deployment challenges.

To address the aforementioned challenges, this paper proposes an PD-YOLO method based on a multi-scale feature fusion network, building on YOLOv8n. The method introduces an innovative Parallel Focusing Feature Pyramid (PF-FPN), which effectively improves the accurate classification and localization of different types of weeds in complex environments. The PF-FPN includes the Feature Filtering and Aggregation Module (FFAM) and the Hierarchical Adaptive Recalibration Fusion Module (HARFM). The FFAM module utilizes deep convolution and attention mechanisms to preliminarily filter and extract features, adaptively adjusting and fusing multi-scale features to capture rich semantic information. This enhances small object detection and multi-scale feature fusion, thereby improving the model’s accuracy and robustness. The HARFM module leverages attention mechanisms to fuse features at different levels, achieving adaptive optimization and enhancing the model’s expressive power. To further improve detection performance, a dynamic detection head (Dyhead) (Dai et al., 2021) is introduced, enhancing the model’s stability and accuracy in complex backgrounds. Ultimately, the proposed PD-YOLO model integrates the PF-FPN network and Dyhead architecture, using YOLOv8n as the base framework. This optimization of feature fusion and model representation capabilities significantly enhances the overall accuracy of weed detection.

The remainder of this paper is organized as follows: Section 2 introduces related research work; Section 3 presents the PD-YOLO model; Section 4 describes the experiments conducted on the model; Section 5 provides relevant discussions; and Section 6 concludes the paper.

## 2 Related works

Early research in weed detection primarily relied on traditional image processing techniques (Wu et al., 2021). These methods involved extracting features such as color, texture, and shape from images, which were then used in conjunction with machine learning algorithms like Random Forests or Support Vector Machines for weed identification (Sabzi et al., 2020). For example, Islam et al (Islam et al., 2021). achieved efficient weed detection in Unmanned Aerial Vehicle (UAV) imagery through image orthorectification combined with machine learning algorithms, reaching accuracy rates of 98.40% on the original dataset and 94.72% on an extended dataset. The success of these techniques heavily depended on the quality of image acquisition, preprocessing, and feature extraction, as these factors directly influenced the performance and generalization ability of the algorithms.

With the advent of deep learning, object detection methods have revolutionized weed detection due to their superior efficiency and accuracy. Tang et al. (2017). pioneered the use of K-means unsupervised feature learning in conjunction with Convolutional Neural Networks (CNNs), improving the identification accuracy of soybean seedlings and associated weeds to 92.89% through fine-tuning optimization. This approach effectively addressed the issues of instability and limited generalization found in manually designed feature-based methods. Similarly, dos Santos Ferreira et al (Tang et al., 2017) applied a ConvNets network to detect weeds in soybean crop images, classifying them into grass and broadleaf categories. This categorization enabled the targeted application of specific herbicides, achieving over 98% identification accuracy.

Currently, classical deep learning-based object detection methods are mainly categorized into single-stage and two-stage detection algorithms. Single-stage algorithms directly use neural networks to extract features from images and perform detection. Representative algorithms include SSD (Liu et al., 2016), the YOLO series (Redmon et al., 2016; Chen et al., 2021), and RT-DETR (Chen et al., 2021). Single-stage algorithms generally have the advantage of faster speed and better real-time detection performance, but they often suffer from lower localization accuracy. In contrast, two-stage algorithms first generate candidate regions and then classify and localize these regions. They usually achieve higher detection accuracy but are relatively slower and require more computational resources, with Faster R-CNN (Ren et al., 2015) being a representative example.

In weed detection, two-stage algorithms first generate potential weed-containing regions using object detection algorithms, followed by deep learning model classification to distinguish weeds from crops. Veeranampalayam Sivakumar et al (Veeranampalayam Sivakumar et al., 2020). constructed Faster R-CNN and SSD models and evaluated their performance for weed detection in soybean fields using UAV images. The results showed that both models performed well in weed detection, but Faster R-CNN outperformed SSD in terms of performance. This approach typically improves detection accuracy and reduces false positive rates, but its high computational complexity makes it difficult to meet real-time requirements.

To address the challenges of morphological diversity, scale variations, and complex backgrounds in farmland weed detection, researchers have proposed various improvements based on the YOLO series models. These methods enhance model performance in specific agricultural scenarios through strategies such as integrating attention mechanisms, optimizing multi-scale feature fusion, and improving small-target detection capabilities. **Table 1** systematically compares representative models in terms of improvement methods, parameter counts, computational costs, and performance metrics.

**Table 1:** Systematic comparison of YOLO-based improvement methods and performance metrics for farmland weed detection tasks.

Model	Application Scenario	Improvement Methods	Parameters (M)	GFLOPS (G)	mAP 0.5 (%)
GTCBS-YOLOv5s (Shao et al., 2023)	Rice field weed identification	YOLOv5 + Ghost + C3Trans + CBAM + BiFPN + SIoU loss	4.63	25.1	91.1
YOLOV7-G (Yu et al., 2024)	Sesame field weed identification	YOLOv7 + SimAM + C3 module + SPPFCSPC + Focal-SIoU loss	0.57	0.48	56.6
YOLO-Riny (Xu et al., 2024)	Herbal field weed tracking	YOLOv7-tiny + FasterNet backbone + lightweight upsampling + ByteTrack	10.1	11.2	91.7
YOLOv8-DMAS (Zheng et al., 2024)	Cotton field weed detection	YOLOv8 + DWR + MSBlock + small-target layer + ASFF + SoftNMS	19.03	51.2	95.5
RMS-DETR (Guo et al., 2024)	Rice field weed identification	DETR + multi-scale feature fusion + global context modeling	40.8	187	85.1
YOLO-CWD (Ma et al., 2025)	Corn-weed detection	YOLOv8 + hybrid attention + PIoU loss	3.49	9.6	75.1

Current YOLO-based farmland weed detection models generally suffer from scenario limitations and methodological homogenization. Most models, such as GTCBS-YOLOv5s (Shao et al., 2023) and YOLOv8-DMAS (Zheng et al., 2024), are optimized solely for single environments like rice or cotton fields, relying on repetitive technical approaches including attention mechanisms, multi-scale feature fusion, and loss function improvements, while lacking differentiated designs for weed morphological diversity and crop coexistence scenarios. Some models like RMS-DETR (Guo et al., 2024) with 40.8M parameters and 187 GFLOPs computational cost achieve only 85.1% accuracy, showing significant efficiency-accuracy imbalance. Lightweight models such as YOLOV7-G (Yu et al., 2024), though compressed to 0.57M parameters, suffer from high missed detection rates resulting in mAP as low as 56.6%, limiting practical applicability. Although a few models like YOLO-Riny (Xu et al., 2024) achieve edge device compatibility through structural lightweighting, their improvements remain confined to specific weed types Corydalis edulis and Setaria viridis in cornfields, failing to address complex multi-target interaction detection needs. While YOLO-CWD (Ma et al., 2025) achieves lightweight design with 75.1% mAP@50 and 9.6 GFLOPS in cornfield weed detection through hybrid attention mechanisms and PIoU loss function, its detection accuracy and model compactness still require further optimization. Existing studies generally lack cross-scenario generalization validation, showing weak support for multi-category weed interaction detection, environmental robustness, and crop-weed coexistence mechanisms, which constrains practical agricultural applications.

Existing weed detection methods based on general deep learning architectures face challenges due to the diversity in weed morphology and environmental conditions, making algorithm development for different plant species difficult (Hu et al., 2024). Additionally, Convolutional Neural Networks (CNNs), while extracting image features, are limited by the local receptive field of convolutional operators, making it hard to capture global information, which affects accurate image localization and classification (Luo et al., 2016). To overcome this limitation, researchers often employ multi-scale feature fusion techniques, with parallel multi-branch networks and serial skip-connection structures being two commonly used approaches.

In the Inception module of GoogLeNet, the parallel multi-branch network extracts multi-scale and hierarchical feature information from the same feature map using convolutional kernels of different sizes (Szegedy et al., 2015). Although this method takes advantage of convolution kernels with different receptive fields and carefully designed modules to learn rich multi-scale features, it overlooks semantic differences between features of different scales, which can lead to the loss of semantic information. High-level feature maps generated by the backbone network contain rich semantic information but lack the detailed information of objects, while low-level features, although containing precise object locations, lack sufficient semantic information.

To address this issue, high-dimensional features are typically upsampled and aligned with downsampled low-dimensional features, followed by pixel-wise summation to enhance semantic information. However, this method does not perform feature selection, merely summing pixel values across multiple feature layers, which may lead to redundant and repetitive information. Consequently, this approach fails to fully integrate and utilize diverse features (Chen et al., 2024).

In addition, feature fusion networks represent an efficient method for multi-scale fusion. The classic Feature Pyramid Network (FPN) (Lin et al., 2017) employs a top-down pyramid structure to achieve multi-scale feature fusion. However, due to its structural characteristics, FPN lacks sufficient high-level semantic information. To enhance local localization information, PANet (Liu et al., . 2018) added a bidirectional feature fusion module on top of FPN. Building on these approaches, BiFPN (Tan et al., 2020) introduced bidirectional connections in the process of information propagation, allowing information to flow both top-down and bottom-up within the network. This bidirectional flow effectively addresses issues of information loss and blurring in feature pyramid networks.

The Gold-YOLO model introduced an advanced gather and distribute mechanism (GD mechanism), which uses a unified module to collect and fuse information from all levels and distribute it to different levels, addressing the information fusion issues in traditional object detection models (Wang et al., 2024). Although multi-scale feature fusion methods have significant reference value for image processing tasks, current networks struggle to meet the practical requirements of weed detection tasks due to challenges such as limited weed features, varying lighting conditions, and occlusion problems. Therefore, developing more efficient feature fusion networks is crucial for improving the accuracy of weed detection.

## 3 Method

### 3.1 PD-YOLO model

**Figure 1:**
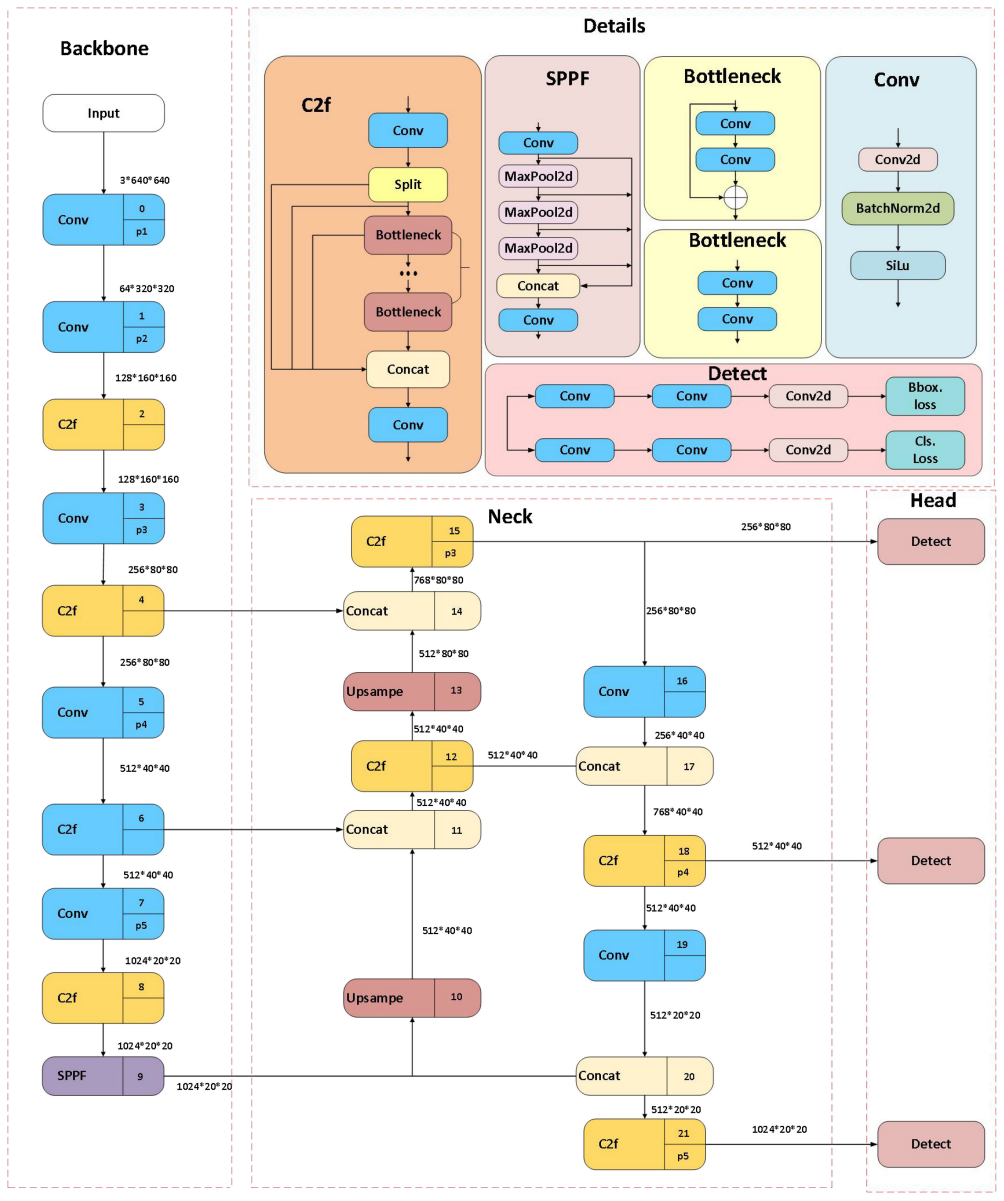
The overall architecture of the YOLOv8 model.

YOLOv8 is an end-to-end optimized model known for its high performance and accuracy in detection and segmentation tasks in computer vision (Redmon et al., 2016; Chen et al., 2021; Reis et al., 2023). It builds upon the improvements made in YOLOv5 (Zhang et al., 2022), and its specific structure is shown in **Figure 1**. The C2f module is a key component of YOLOv8’s backbone network, enhancing the richness of information flow through gradient-splitting connections while maintaining a lightweight model. The neck part uses a PA-FPN structure, inspired by PANet (Liu et al., . 2018), and the head adopts a decoupled structure designed separately for object classification and bounding box regression, utilizing different loss functions to improve detection accuracy and model convergence speed. This design, combined with a dynamic sample allocation mechanism, further enhances YOLOv8’s detection accuracy and robustness.

The YOLOv8 series includes multiple versions, offering more refined model parameter tuning options, making it highly effective in both high-precision and real-time applications. YOLOv8n (Nano) is the smallest and fastest version of the YOLOv8 series, suitable for mobile devices, embedded systems, and applications that require real-time processing. Its efficient performance in terms of processing speed and computational resources makes it ideal for weed detection applications.

**Figure 2:**
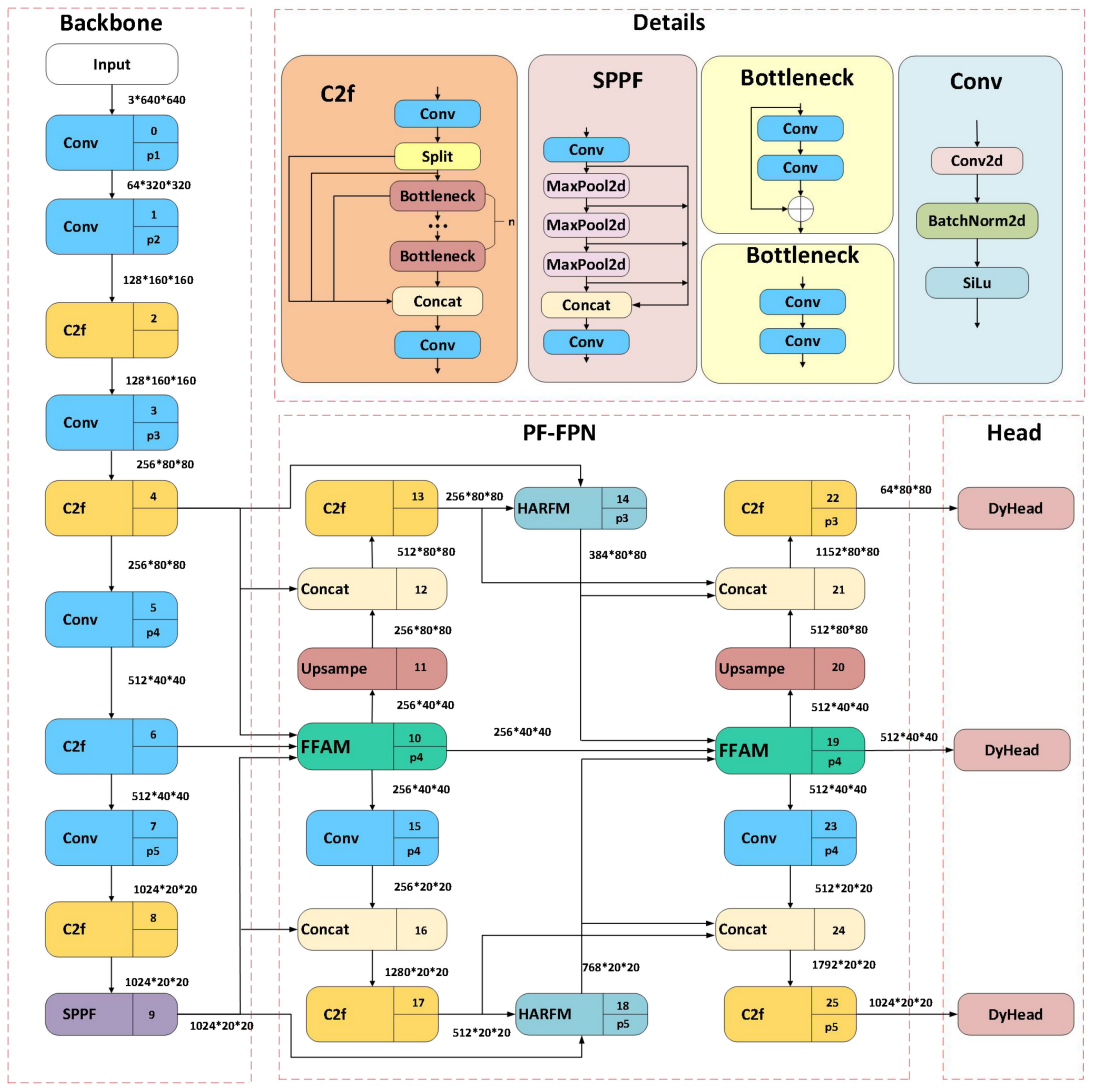
Overall architecture of FD-YOLO. PF-FPN is the feature fusion network proposed in this study, which fuses three scale features from the backbone network based on the FAFM module. Meanwhile, the HARFM module fuses low-level and high-level features within the same path and aggregates them into the FFAM module.

Based on the lightweight YOLOv8n model, we designed an improved weed detection model—PD-YOLO. The structure of PD-YOLO is shown in **Figure 2**, and it primarily enhances the accuracy and efficiency of weed detection through the organic combination of three key components: the Backbone, the Parallel Focusing Feature Pyramid (PF-FPN), and the DyHead.

(1) The Backbone utilizes multiple convolutional layers to extract multi-scale features from the input image, and the C2F module enhances the feature map’s expressive capability, effectively capturing and integrating information from different levels to support efficient and accurate object detection tasks.
(2) The PF-FPN is a multi-layer feature fusion pyramid that enables efficient multi-scale feature fusion, addressing the issue of similar features between weeds and plants as well as between different weeds, thereby improving the detection capability of various weed types. PF-FPN includes the Feature Filtering and Aggregation Module (FFAM) and the Hierarchical Adaptive Recalibration Fusion Module (HARFM). The FFAM module first filters and fuses features, and then extracts them, achieving multi-scale feature extraction. The HARFM module, based on attention mechanisms, further enhances feature expression, effectively improving the model’s feature fusion capability.
(3) The detection head is responsible for object localization and classification based on the fused features. In field environments, weeds exhibit diverse scales and complex morphologies, which increases the difficulty of detection. To tackle these challenges, this study introduces dynamic head technology, which adaptively adjusts the parameters and structure of the detection head to more effectively capture information from different feature layers, enhancing the model’s adaptability to complex scenarios.

### 3.2 Parallel focusing feature pyramid

**Figure 3:**
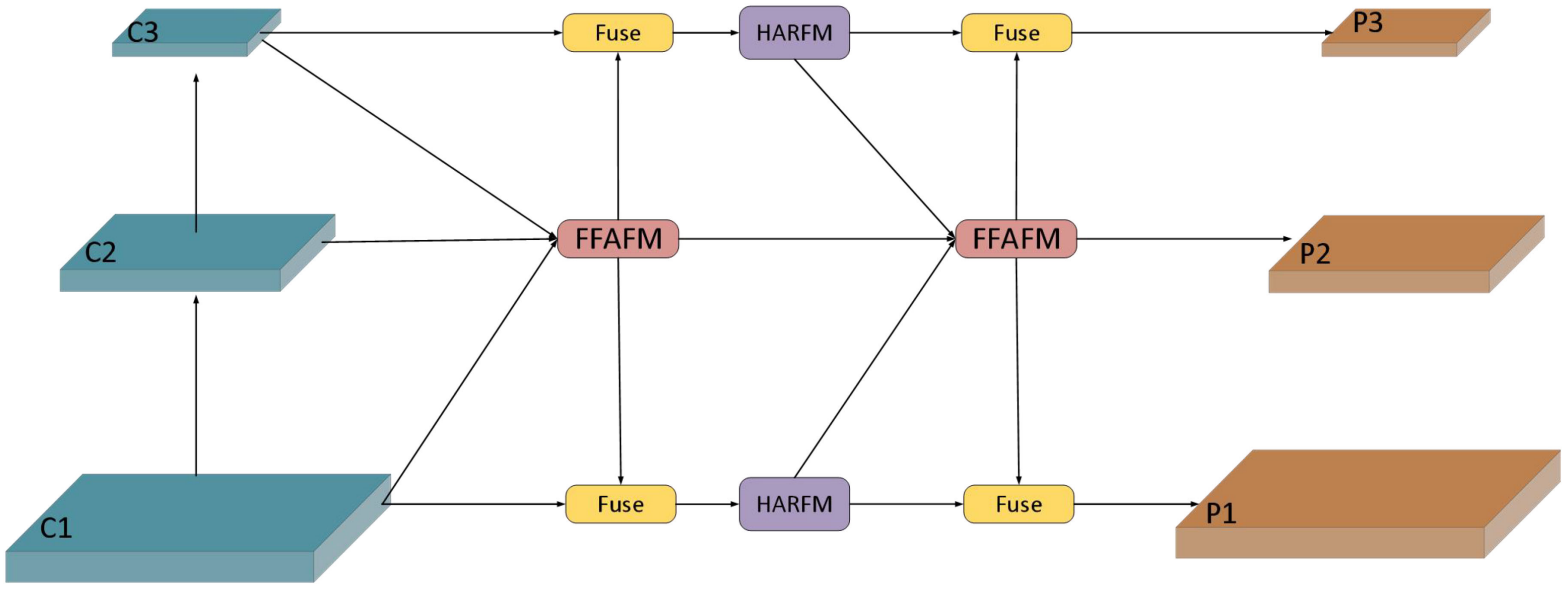
The structure of the Parallel Focusing Feature Pyramid.

The structure of PF-FPN is shown in **Figure 3**. It consists of two parts: the FFAM module and the HARFM module. “Fuse” represents the process of fusing multi-scale features. The features C1, C2, and C3 are derived from different levels of the backbone network, representing information at various scales: C1 originates from lower levels, containing high resolution and rich details; C2 comes from the intermediate levels, balancing resolution and semantic information; and C3 comes from the higher levels, containing deeper semantic information despite lower resolution. The {C1, C2, C3} features are aggregated into the FFAM module, where efficient multi-scale feature fusion is achieved through attention mechanisms and convolutional layers. The fused features are then distributed across various detection scales through convolutional Downsampling or Upsampling, concatenated with features of the same level, and passed to the C2F module in **Figure 2** for further fusion. The HARFM module fuses low-level and high-level features along the same path before aggregating them into the FFAM module. This parallel feature fusion, from left to right and from the center to the edges, produces the final {P1, P2, P3} feature layers, achieving complementary enhancement of multi-level features. This parallel dynamic feature fusion mechanism takes into account the diversity of feature hierarchies and effectively avoids information loss and bias through parallel fusion, significantly improving the model’s ability to handle large-scale variations and similar feature targets. Compared to the traditional FPN, which uses a unidirectional top-down feature fusion path, this parallel fusion approach better preserves the detailed information of low-level features, avoiding the loss of detail features that may occur in traditional methods.

### 3.3 Feature filtering and aggregation module

**Figure 4:**
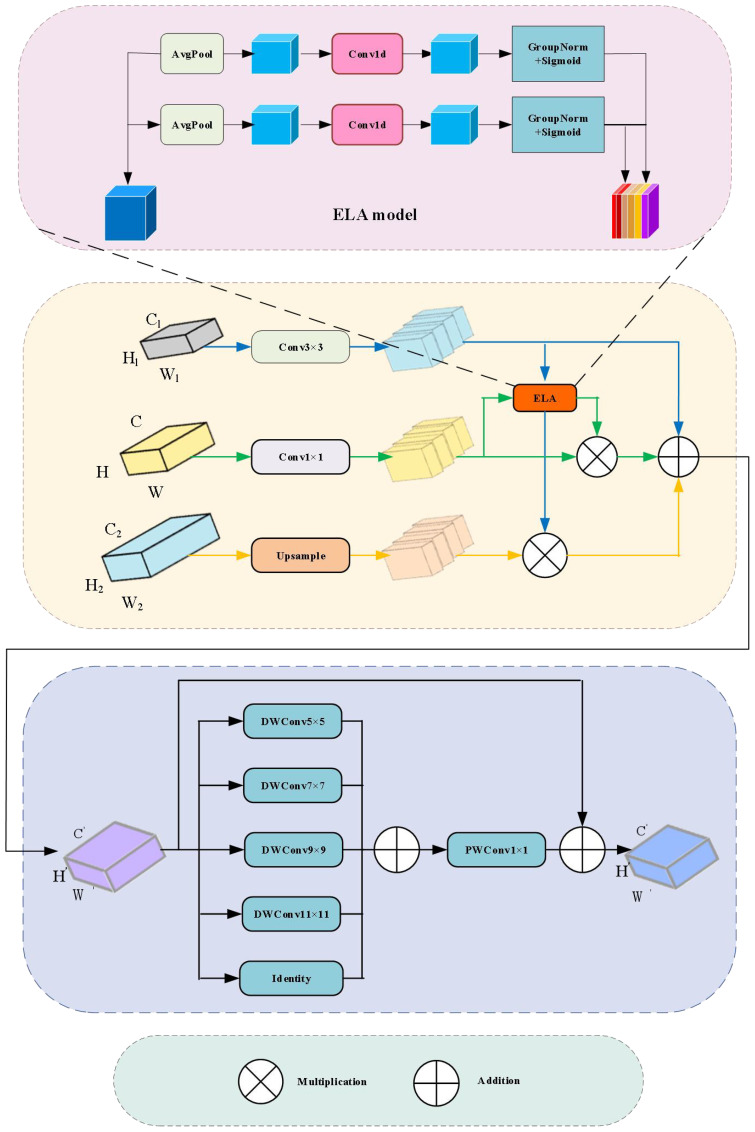
Feature Filtering and Aggregation Module.

During feature extraction, unfiltered features often introduce noise and redundant information, which can negatively impact subsequent analysis and decision-making processes. Therefore, a method of filtering before extraction is proposed. The structure of the FFAM module is shown in **Figure 4**.

The filtering step for multi-scale features benefits from the ELA attention mechanism (Xu and Wan, 2024), which can dynamically adjust and filter based on the characteristics of the input data. It helps suppress background noise, such as soil textures, and enhances small target regions like weed leaves. Compared to SE attention (Hu et al., 2018), which only focuses on channel relationships, ELA is more suitable for agricultural scenarios with irregular spatial distributions. The feature extraction method involves using a set of parallel depthwise separable convolutions to extract multi-scale detailed features from the filtered and fused features. This channel fusion mechanism helps integrate features with different receptive field sizes, capturing extensive contextual information.

The input feature map F of the module consists of three features at different scales: a low-level feature ${\text{F}}_{\text{low}}\in {\text{ R}}^{\text{H}1\times \text{W}1\times \text{C}1}$ , a high-level feature ${\text{F}}_{\text{high}}\in {\text{ R}}^{\text{H}2\times \text{W}2\times \text{C}2}$ , and a mid-level feature ${\text{F}}_{\text{mid}}\in {\text{ R}}^{\text{H}\times \text{W}\times \text{C}}$ .The mathematical expression for this process is as follows, as shown in Equation 1:


(1) $$\begin{array}{c} {\text{F}}_{\text{low}}^{\text{ "}}={\text{f}}_{2\text{d}}({\text{F}}_{\text{low}})\\ \begin{array}{c} {\text{F}}_{\text{mid}}^{\text{ "}}={\text{f}}_{2\text{D}}({\text{F}}_{\text{mid}})\\ {\text{F}}_{\text{high}}^{\text{ "}}={\text{f}}_{\text{up}}({\text{F}}_{\text{high}}) \end{array} \end{array}$$

Where ${\text{f}}_{2\text{d}}$ denotes 2D convolution and; ${\text{f}}_{2\text{D}}$ denotes downsampling using a 2D convolution with a kernel size of 3 and a stride of 2; ${\text{f}}_{\text{up}}$ denotes upsampling. On this basis, the high-level feature ${\text{F}}_{\text{high}}^{\text{ "}}$ generates the corresponding attention weight through the ELA attention mechanism, which is used to filter the low-level features. Meanwhile, the mid-level feature ${\text{F}}_{\text{mid}}^{\text{ "}}$ also generates corresponding attention weights through the ELA module to filter its own redundant information. Subsequently, the filtered multi-scale features are added and fused to obtain MS∈RH'×W'×C' . The mathematical expression for this process is as follows, as shown in Equation 2:


(2) $${\text{M}}_{\text{S}}={\text{f}}_{\text{ELA}}({\text{F}}_{\text{high}}^{\text{ "}})\times {\text{F}}_{\text{low}}^{\text{ "}}+{\text{F}}_{\text{high}}^{\text{ "}}+{\text{f}}_{\text{ELA}}({\text{F}}_{\text{mid}}^{\text{ "}})\times {\text{F}}_{\text{mid}}^{\text{ "}}$$

Where ${\text{f}}_{\text{ELA}}$ represents the attention weights generated by the ELA module. The ELA attention mechanism is crucial in the FFAM module, as shown in **Figure 4**. ELA is a novel attention mechanism that uses a simple and lightweight structure, enabling the network to precisely focus on regions of interest. It first uses adaptive average pooling to pool the input feature map x vertically and horizontally, with horizontal direction as (1, H) and vertical direction as (W, 1). For the ${\text{c}}^{\text{th}}\;$ channel, the height h and width w are expressed as shown in Equations 3 and 4:


(3) $${\text{Z}}_{c}^{\text{h}}(\text{h})=\frac{1}{\text{H}}\;\sum_{0\leq \text{i}<\text{w}}{\text{x}}_{\text{c}}(\text{h},\text{i})$$

(4) $${\text{Z}}_{c}^{\text{w}}(\text{w})=\frac{1}{\text{w}}\sum_{0\leq \text{j}<\text{H}}{\text{x}}_{\text{c}}(\text{j},\text{w})$$

The two obtained feature vectors, ${\text{Z}}_{c}^{\text{h}}$ and ${\text{Z}}_{c}^{\text{w}}$ , are respectively processed through a 2D convolution, followed by a group normalization layer (GN), and finally a Sigmoid activation function to generate the attention weights. The process is illustrated in Equations 5 and 6:


(5) $${\text{f}}_{\text{h}}=\text{σ}({\text{f}}_{\text{GN}}({\text{f}}_{2\text{d}}(Z_{c}^{h}\;))$$

(6) $${\text{f}}_{\text{w}}=\text{σ}({\text{f}}_{\text{GN}}({\text{f}}_{2\text{d}}(Z_{c}^{w}\;))$$

where ${\text{f}}_{2\text{d}}$ denotes 2D convolution, ${\text{f}}_{\text{GN}}$ denotes group normalization with 16 groups, and σ denotes the Sigmoid function.The horizontal and vertical outputs are multiplied to obtain the resulting attention weights, represented as shown in Equation 7:


(7) $${\text{f}}_{\text{ELA}}={\text{f}}_{\text{h}}\times {\text{f}}_{\text{w}}$$

On the basis of initially filtered features, the module employs a set of parallel depthwise separable convolutions to extract multi-scale detailed features, enhancing the capability to capture small targets and rich semantic information, thus improving the model’s generalization and robustness. Inspired by PKI (Cai et al., 2024), an Inception-style feature extraction module (Yu et al., 2024) is introduced, as shown in **Figure 4**, which demonstrates good performance in handling multi-scale target detection tasks. The large convolutional kernels can recognize larger weed shapes, while the small convolutional kernels focus on small target weeds. Compared to the single-scale convolutions in the BiFPN network, this design is more flexible in adapting to the scale variations of weed shapes. The module uses a set of parallel depthwise separable convolutions to capture small target features and contextual semantic information, with direct connections added. Specifically, according to the parameter settings of the PKI module, the optimal kernel size for the ${\text{m}}^{\text{th}}$ DWConv is set to: $k^{\text{m}}=(\text{m}+1)\times 2+1$ .The module uses a total of 4 DWConv layers, with kernel sizes of 5, 7, 9, and 11. These are followed by a 1×1 pointwise convolution (Pwconv) to fuse the local and contextual features, generating the output feature ${\text{M}}_{\text{C}}\in {\text{R}}^{{\text{H}}^{\prime }\times {\text{W}}^{\prime }\times {\text{C}}^{\prime }}$ . The expression for ${\text{M}}_{\text{C}}$ is as follows:


(8) $${\text{M}}_{\text{C}}={\text{f}}_{\text{p}}(\;\sum_{1\leq \text{m}\leq 4}{\text{f}}_{{\text{D}}_{{\text{K}}^{m}}}({\text{M}}_{\text{S}})\;+{\text{M}}_{\text{S}})$$

Where ${\text{f}}_{{\text{D}}_{{\text{K}}^{m}}}$ the ${\text{m}}^{\text{th}}$ 3×3 depthwise separable convolution; ${\text{f}}_{\text{p}}$ represents the pointwise convolution. Finally, ${\text{M}}_{\text{C}}$ is combined with the original input feature ${\text{M}}_{\text{S}}$ to obtain the rich semantic output feature $\text{F"}\in {\text{R}}^{{\text{H}}^{\prime }\times {\text{W}}^{\prime }\times {\text{C}}^{\prime }}$ . The expression is given in Equation 9:


(9) $${\text{F}}^{\prime }={\text{M}}_{\text{S}}(\text{F})+{\text{M}}_{\text{C}}({\text{M}}_{\text{S}}(\text{F}))$$

### 3.4 Hierarchical adaptive recalibration fusion module

**Figure 5:**
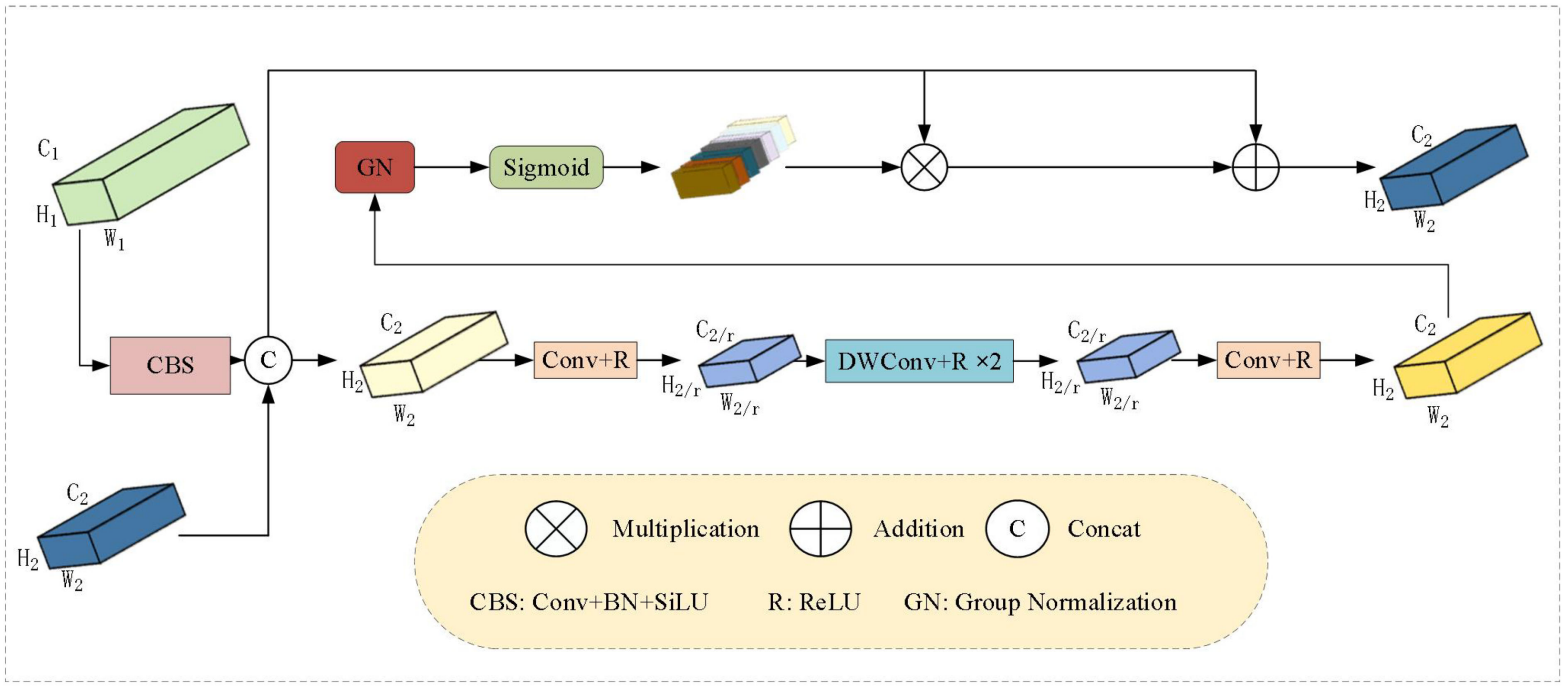
HARFM structure.

In the feature pyramid structure, high-level features contain rich semantic information due to their deep receptive fields but have lower spatial resolution, while low-level features retain high-resolution detail information but lack global semantic context. The traditional Feature Pyramid Network (FPN) fuses multi-scale features through simple linear summation, which can dilute semantic information, especially making it less sensitive to low-contrast overlapping leaf regions. Therefore, the HARFM module recalibrates the attention weights obtained by concatenating high-level and low-level features, enhancing the network’s focus on key features and improving the overall performance of weed detection. Specifically, group normalization (GN) is used instead of batch normalization (BN) to avoid the statistical bias issues during small-batch training, which is crucial for agricultural images with complex data distributions. The HARFM structure is shown in **Figure 5**.

The low-level feature ${\text{X}}_{\text{l}}\in {\text{ R}}^{{\text{C}}_{1}\times {\text{H}}_{1}\times {\text{W}}_{1}}$ and the high-level feature ${\text{X}}_{2}\in {\text{ R}}^{{\text{C}}_{2}\times {\text{H}}_{2}\times {\text{W}}_{2}}$ have a large difference in the number of channels, which would result in high computational cost if concatenated directly. Therefore, the input low-level feature ${\text{X}}_{\text{l}}$ is passed through a 3×3 convolution to reduce the number of channels to ${\text{C}}_{2}$ , making its dimensions consistent with ${\text{X}}_{2}$ , and then they are concatenated to obtain a feature map ${\text{F}}_{\text{c}}$ in 2 ${\text{C}}_{2}\times {\text{H}}_{2}\times {\text{W}}_{2}$ .The process is represented as shown in Equation 10:


(10) $${\text{F}}_{\text{C}}={\text{f}}_{\text{C}}({\text{f}}_{\text{CBS}}({\text{X}}_{\text{l}}),{\text{X}}_{2})$$

where ${\text{f}}_{\text{CBS}}$ represents the low-level feature processed by 2D convolution, batch normalization (BN), and activation function (Silu). The function ${\text{f}}_{\text{C}}$ denotes concatenation. The concatenated features are then passed through a series of convolutions to generate the attention weights. To reduce computational cost, the concatenated feature is first passed through a 2D convolution to reduce the number of channels to 2 ${\text{C}}_{2}/\text{r}$ , resulting in ${\text{F}}_{1}\in {\text{ R}}^{{\text{C}}_{2}/\text{r}\times {\text{H}}_{2}\times {\text{W}}_{2}}$ , expressed as Equation 11:


(11) $${\text{F}}_{1}=\text{δ}({\text{f}}_{2\text{d}}({\text{F}}_{\text{C}}))$$

where ${\text{f}}_{2\text{d}}$ represents 2D convolution and δ represents the ReLU activation function. Then, two depthwise separable convolutions are used to effectively extract low-level features while reducing the number of parameters: The process is represented as shown in Equation 12:


(12) $${\text{F}}_{2}=\text{δ}({\text{f}}_{\text{D}}(\text{δ}({\text{f}}_{\text{D}}({\text{F}}_{1})))$$

where ${\text{f}}_{\text{D}}$ represents depthwise separable convolution. Finally, a 1×1 convolution is applied to restore the channel number to $2{\text{C}}_{2}$ , resulting in ${\text{F}}_{3}\in {\text{ R}}^{2{\text{C}}_{2}\times {\text{H}}_{2}\times {\text{W}}_{2}}$ . The process is represented as shown in Equation 13:


(13) $${\text{F}}_{3}=\text{δ}({\text{f}}_{2\text{d}}({\text{F}}_{2}))$$

The ReLU activation function is introduced to improve computational efficiency. Finally, the feature map is processed through a Group Normalization (GN) layer and a Sigmoid function to generate the attention weights. The output is obtained by multiplying the original features with the weight matrix and then adding them together. The mathematical expression is given as Equation 14:


(14) $${\text{F}}_{\text{out}}=\text{σ}({\text{f}}_{\text{GN}}({\text{F}}_{3}))\times {\text{F}}_{\text{C}}+{\text{F}}_{\text{C}}$$

### 3.5 Dynamic head

**Figure 6:**
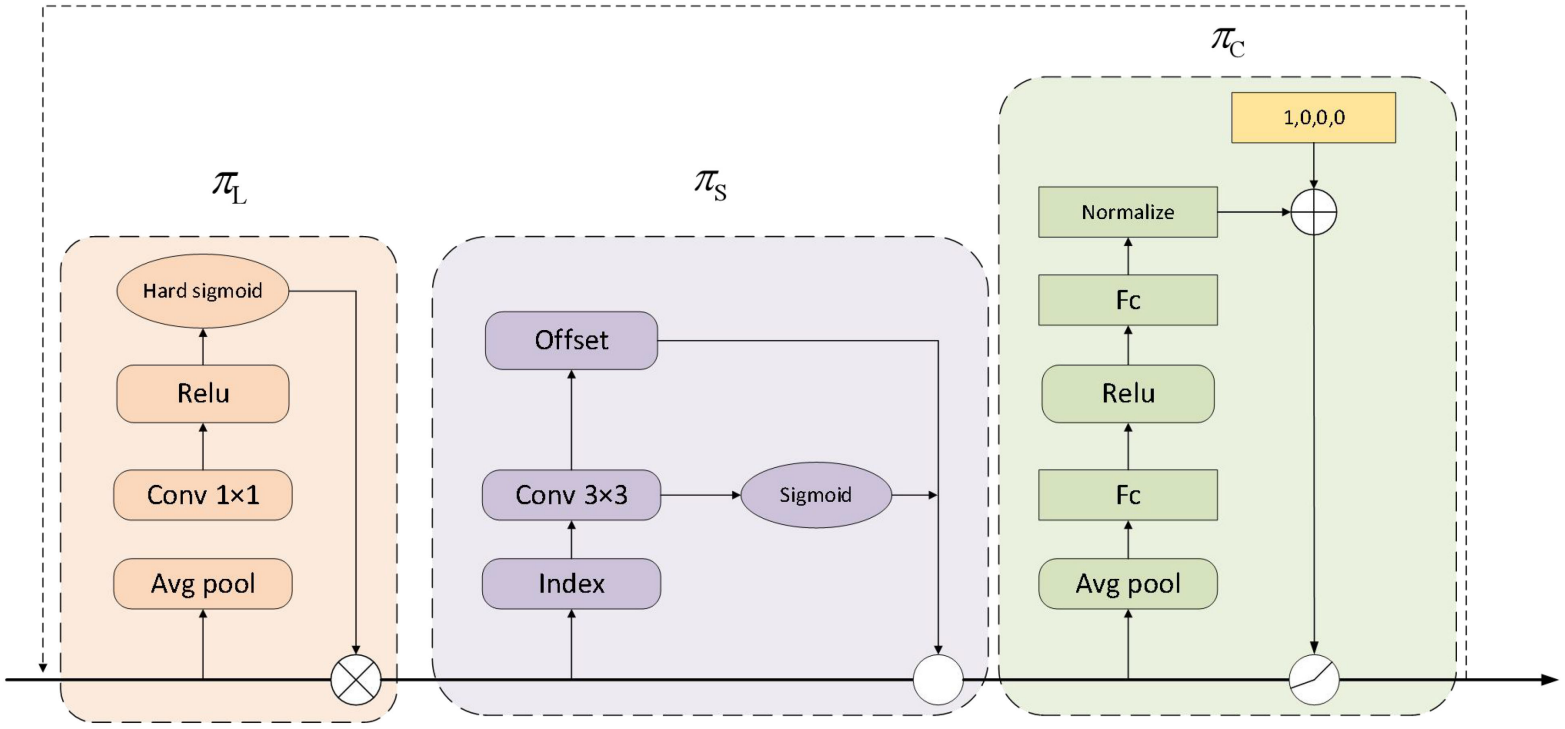
The detailed design of the Dynamic Head and the structure of each attention module.


The structure of the Dynamic Head is illustrated in **Figure 6**. The Dynamic Head incorporates multiple attention mechanisms: Scale-aware, Spatial-aware, and Task-aware. This design enables the Dynamic Head to address scale variations, spatial changes, and different task requirements, thereby improving the efficiency and accuracy of weed detection. Specifically, multi-level features from the feature pyramid are adjusted to the same scale and reshaped into three-dimensional tensors. The attention mechanism is applied according to the formula in Equation 15:

(15) W(F)=π(F)·F

where π represents the attention function, implemented through fully connected layers. The attention mechanism operates along three dimensions, with each attention mechanism focusing only on a specific dimension to ensure computational efficiency. The attention function is defined as in Equation 16:


(16) $$\text{W}(\text{F})={\text{π}}_{\text{C}}({\text{π}}_{\text{S}}({\text{π}}_{\text{L}}(\text{F})\cdot \text{F})\cdot \text{F})\cdot \text{F}$$

Where ${\text{π}}_{\text{L}}$ , ${\text{π}}_{\text{S}}$ , and ${\text{π}}_{\text{C}}$ are three distinct attention functions applied to dimensions L, S, and C respectively.

The description of the multiple attention mechanisms—Scale-aware, Spatial-aware, and Task-aware—is as follows:

#### 3.5.1 Scale-aware attention

The Scale-aware Attention module is designed to handle targets of different scales by distinguishing the relative importance between feature layers and dynamically adjusting feature representations to adapt to various target scales. The input features first go through an average pooling layer to reduce the number of parameters, then are passed through a convolution layer with a kernel size of 1, using the ReLU activation function to better capture non-linear relationships in the input data. Finally, a Sigmoid activation function is applied to produce the final output. The mathematical expression is as follows in Equation 17:


(17) $${\text{π}}_{\text{L}}(\text{F})\cdot \text{F}=\text{σ}(\text{f}(\frac{1}{\text{SC}}\sum_{\text{S},\text{c}}\text{F}))\cdot \text{F}$$

where F represents the input feature tensor, F is a linear function approximating a 1×1 convolution, and σ represents the Sigmoid activation function.

#### 3.5.2 Spatial-aware attention

The Spatial-aware Attention module is designed to capture the spatial consistency of targets. This module enhances the understanding of weed locations and shapes in complex environments by identifying consistent regions across spatial positions and feature hierarchies. To reduce the dimensionality of high-dimensional features, the module operates in two steps: first, deformable convolutions are applied to achieve sparse attention learning, followed by aggregation of feature information from different levels at the same spatial locations. The mathematical expression is shown as follows in Equation 18:


(18) $${\text{π}}_{\text{S}}(\text{F})\cdot \text{F}=\frac{1}{\text{L}}\sum_{\text{l}=1}^{\text{L}}\sum_{\text{k}=1}^{\text{K}}{\text{W}}_{\text{l},\text{k}}\cdot \text{F}(\text{l};{\text{p}}_{\text{k}}+\Delta {\text{p}}_{\text{k}};\text{c}\cdot \Delta {\text{m}}_{\text{k}})$$

In this context, L represents the number of feature layers, and K denotes the number of sparse sampling positions. The term ${\text{p}}_{\text{k}}$ + $\Delta {\text{p}}_{\text{k}}$ indicates the spatial offset that is self-learned to focus on a distinct region, while $\Delta {\text{m}}_{\text{k}}$ reflects the self-learned importance scalar at a specific location, ${\text{p}}_{\text{k}}$ . Both $\Delta {\text{p}}_{\text{k}}$ and $\Delta {\text{m}}_{\text{k}}$ k are derived from the median-level input features of F.

#### 3.5.3 Task-aware attention

The Task-aware attention module adapts to different detection tasks. Specifically, the input feature map x is first passed through an average pooling layer to reduce feature dimensions. Then, two fully connected layers and a normalization layer map the features into the range of -1 to 1. The normalized results are fed into a hyperfunction for further computation. This design enhances the model’s adaptability and performance across various detection scenarios. The mathematical expression is as follows in Equation 19:


(19) $${\text{π}}_{\text{C}}(\text{F})\cdot \text{F}=\text{max}({\text{α}}^{1}(\text{F})\cdot {\text{F}}_{\text{C}}+{\text{β}}^{1}(\text{F}),{\text{α}}^{2}(\text{F})\cdot {\text{F}}_{\text{C}}+{\text{β}}^{2}(\text{F}))$$

where ${\text{F}}_{\text{C}}$ represents the feature slice of the ${\text{c}}^{\text{th}}$ channel, $\left[{\text{α}}^{1},{\text{α}}^{2},{\text{β}}^{1},{\text{β}}^{2}]{\;}^{\text{T}}=\text{θ}(\cdot \right)$ is as a meta-function that learns to control activation thresholds through dimension reduction, neural network layers, normalization, and sigmoid transformation.

## 4 Experiments

### 4.1 Datasets

In this study, two widely varying and challenging datasets, CottonWeedDet12 (Dang et al., 2023) and Lincoln beet (Salazar-Gomez et al., 2021), were chosen instead of a single weed dataset or datasets from specific environments. This approach comprehensively tests the detection capabilities of the PD-YOLO model under different environments and conditions, validating the generality, effectiveness, and robustness of PD-YOLO in real-world applications.

**Figure 7:**
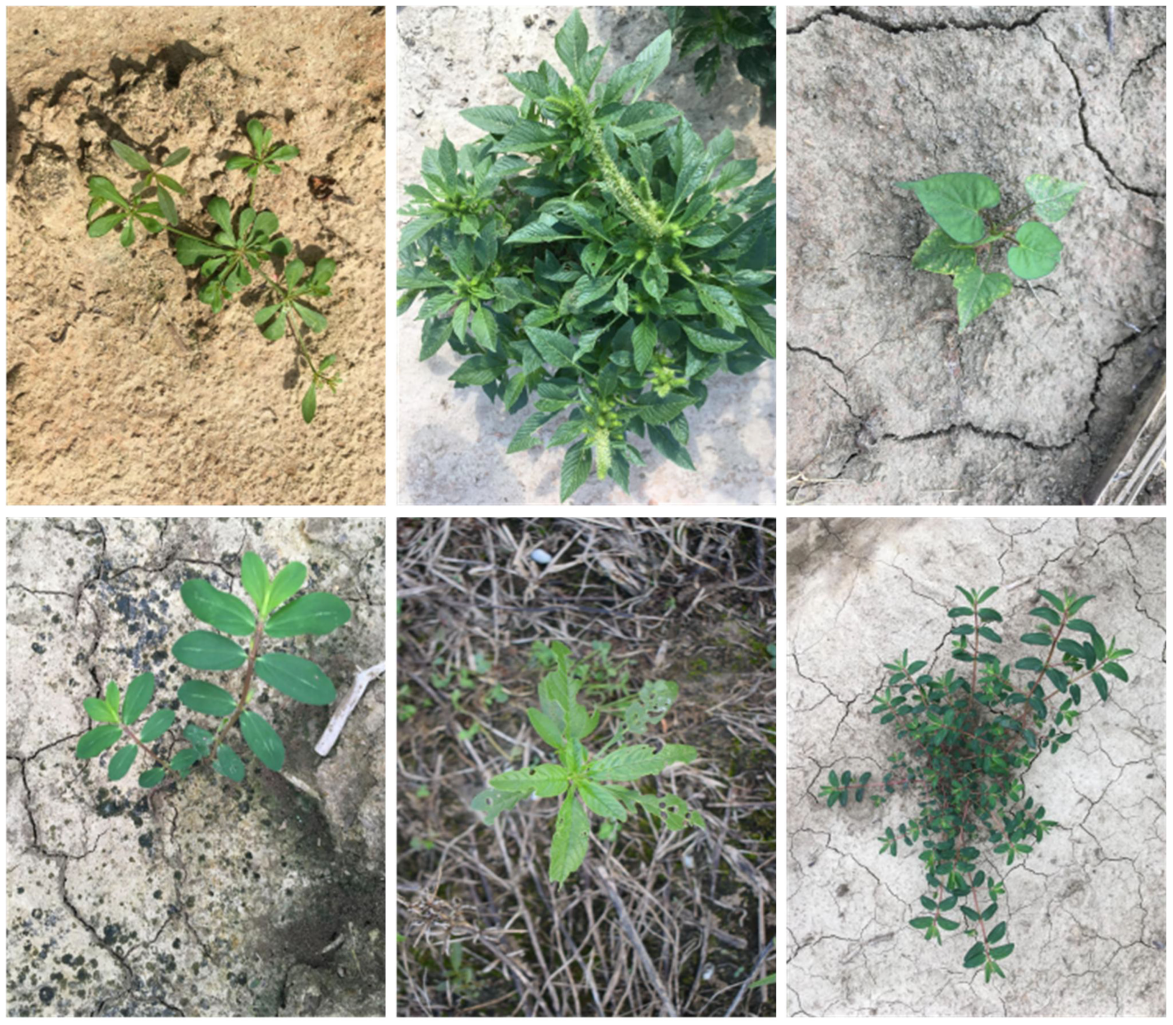
Illustration of images from the CottonWeedDet12 dataset.

**Figure 8:**
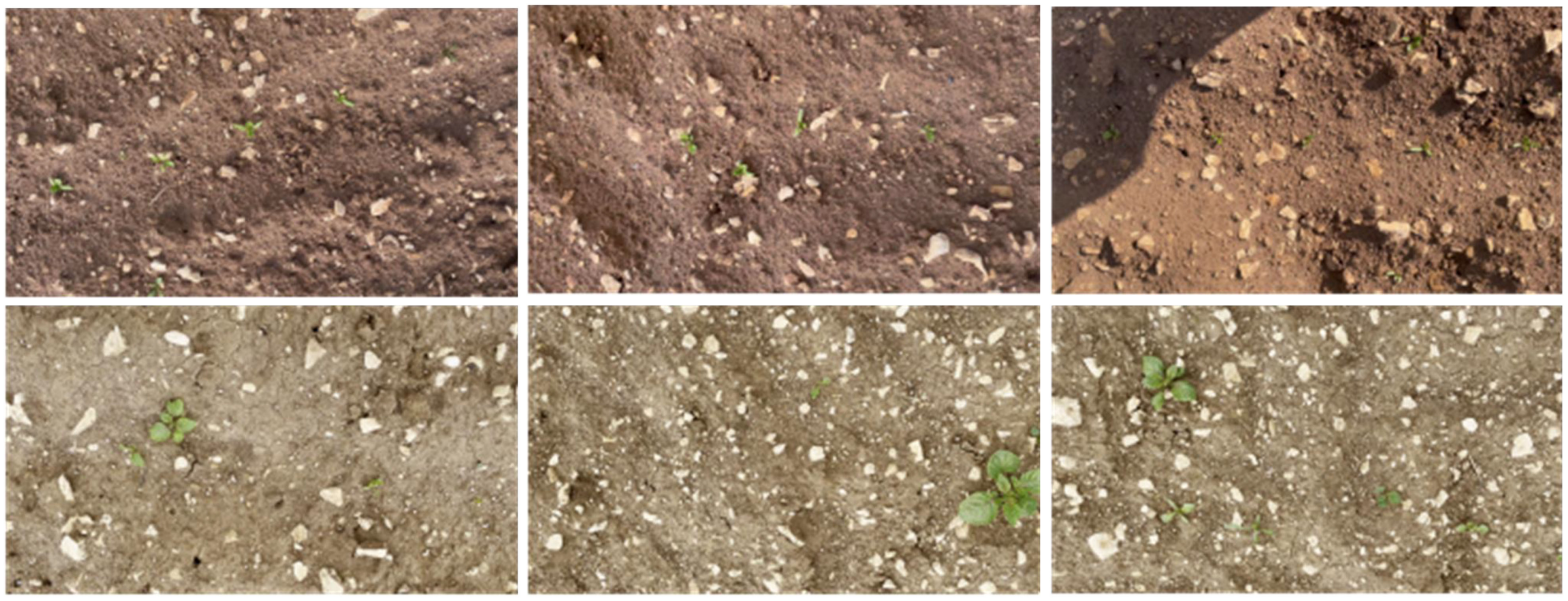
Illustration of images from the Lincoln beet dataset.

(1) CottonWeedDet12 is one of the largest publicly available multi-class weed detection datasets. The dataset covers 12 common weed species found in cotton fields in southern U.S. states, containing 5648 RGB images annotated for weed identification using the VGG Image Annotator (version 2.10), with a total of 9370 bounding boxes. These images were collected under natural field lighting conditions using smartphones or handheld digital cameras from June to September 2021. The dataset is characterized by weed occlusion, large scale differences, and small targets. **Figure 7** shows some original images from the CottonWeedDet12 dataset.

(2) Lincoln beet is a dataset designed for beet and weed detection, specifically focused on addressing the challenge of occlusion. The dataset contains 4405 images with a resolution of 1902×1080 pixels, and each image is annotated with bounding boxes for both beets and harmful weeds, totaling 39,246 bounding boxes. It is a dense dataset with small targets. These images were extracted from videos recorded in different fields in Lincoln, UK. The video recordings were conducted between May and June 2021, using two cameras, with each beet field scanned at least four times per week to capture weed development at various growth stages, showcasing different soil types, plant distributions, and weed species. **Figure 8** shows some original images from the Lincoln beet dataset.

Although the experiments focus on cotton and beet field scenarios, the multi-scale features and weed morphology similarities of the CottonWeedDet12 dataset, along with the high density, occlusion, and small object challenges of the Lincoln Beet dataset, are highly representative and can validate the generalizability of FD-YOLO in complex agricultural environments. Additionally, the PF-FPN design concept of FD-YOLO gives it a certain level of cross-crop adaptability. The global semantic information and local detail features captured by PF-FPN can be generalized to weed detection tasks in other crops (such as corn and wheat). However, the morphological differences, planting densities, and background complexities of different crops may affect model performance, requiring further validation and optimization across datasets.

### 4.2 Performance experiment of PD-YOLO

The experiments were conducted on a 64-bit Windows 11 operating system using an NVIDIA GeForce RTX 3050Ti GPU with 16GB of memory. The PD-YOLO model was implemented in a deep learning environment using Python 3.9.16, torch 2.2.0, and CUDA 12.1. The input image size for the model was set to 640×640, and the model was trained for 200 epochs. During training, mosaic data augmentation was applied, but it was turned off after the 15th epoch. The learning rate was set to 0.01, weight decay to 0.0005, and momentum to 0.937. The CottonWeedDet12 dataset was split in an 8:1:1 ratio for training, validation, and testing, while the Lincoln beet dataset was split in a 7:1:2 ratio.

We conducted experiments comparing PD-YOLO with Faster R-CNN (Ren et al., 2015), SSD (Liu et al., 2016), Yolov7-tiny (Wang et al., 2023), Yolov8n, Yolov8s, Yolov10 (Wang et al., 2024), and RT-DETR (Zhao et al., 2024) to evaluate the performance of PD-YOLO. The experiments were conducted using the CottonWeedDet12 and Lincoln beet datasets, with precision, recall, mAP@0.5, mAP@0.5:0.95, parameters, and FLOPs as evaluation metrics.

(1) Precision, as follows in Equation 20, measures the accuracy of the model when predicting positive classes, which is the ratio of correctly predicted positive samples to all samples predicted as positive.


(20) $$Precision=\;\frac{TP}{TP+FP}$$

Where TP (True Positive) refers to correctly identified positive samples, and FP (False Positive) refers to incorrectly identified negative samples as positive.

(2) Recall, as follows in Equation 21 evaluates the model’s ability to identify positive samples, which is the ratio of correctly predicted positive samples to all actual positive samples.


(21) $$Recall=\frac{TP}{TP+FN}$$

Where FN (False Negative) refers to the positive samples missed by the model.

(3) mAP, as follows in Equation 22, is a core evaluation metric in object detection. It calculates the Average Precision (AP) for each class, then averages them to comprehensively evaluate the model’s detection accuracy, taking into account different classes and various IoU thresholds. The mathematical expression is as follows:


(22) $$\left\{ \begin{array}{c} AP=\int_{0}^{1}\text{p}(\text{r})\text{dr}\\ Map=\frac{1}{\text{C}}\sum_{\text{j}=1}^{\text{c}}{\text{Ap}}_{\text{i}} \end{array}\right.$$

Where c represents the number of classes, and ${\text{Ap}}_{\text{i}}$ represents the average precision for the i class.

(4) FLOPs measure the hardware performance and algorithmic complexity, while FPS represents detection speed by measuring the number of frames processed per second.

The experimental results on the CottonWeedDet12 are shown in **Table 2**. PD-YOLO demonstrates a high precision (P) of 94.3%, outperforming all other models. Its recall (R) reached 87.0%, slightly lower than Yolov8s’s 90.6%, but still excellent at 87.0%, showcasing its superior ability to identify targets. To evaluate detection performance under different IoU thresholds, we used the mean average precision (mAP). The results show that PD-YOLO achieved an mAP@0.5 of 95.0%, the best among all models, surpassing Faster-RCNN by 27.9%. When compared to other high-performance models such as Yolov10 and RT-DETR, PD-YOLO outperformed them by 2.3% and 2.5%, respectively. The mAP@0.5-0.95 was 88.3%, proving its ability to maintain high detection accuracy across different IoU thresholds, highlighting its strong generalization and robustness.

**Table 2:** Performance experiment of PD-YOLO on the CottonWeedDet12 dataset.

Model	Precision (%)	Recall (%)	mAP 0.5 (%)	mAP 0.5-0.95 (%)	FPS
Faster-RCNN	67.1	78.6	67.1	63.9	7.05
SSD	71.9	68.8	71.9	65.0	49.9
Yolov7-tiny	92.5	88.6	94.0	84.1	102.3
Yolov8n	93.8	87.6	93.3	86.5	**109.7**
Yolov8s	89.8	**90.6**	94.2	87.1	87.9
RT-DETR	90.1	90.3	92.5	85.9	31.8
Yolov10s	89.7	86.7	92.7	86.5	72.4
**PD-YOLO**	**94.3**	87.0	**95.0**	**88.3**	42.5

The bold values in the table indicate the optimal performance of each method

The experimental results on the Lincoln beet dataset are shown in **Table 3**. Compared with the baseline model Yolov8n, PD-YOLO achieved improvements of 0.6% in precision (P), 1% in recall (R), 1.3% in mAP@0.5, and 0.9% in mAP@0.5:0.95, demonstrating that the optimizations in PD-YOLO effectively enhance model performance. PD-YOLO outperforms most comparison models in both precision and recall. Specifically, PD-YOLO achieved a precision of 75.4%, 3.9% higher than Faster-RCNN, 13.1% higher than SSD, and slightly higher than Yolov7-tiny. In terms of recall, PD-YOLO reached 71.4%, equal to Yolov10s and 3.6% higher than Faster-RCNN.

**Table 3:** Performance experiment of PD-YOLO on the Lincoln beet dataset.

Model	Precision (%)	Recall (%)	mAP 0.5 (%)	mAP 0.5-0.95 (%)	FPS
Faster-RCNN	71.5	67.8	71.4	49.8	6.90
SSD	62.3	63.0	62.3	39.5	49.9
Yolov7-tiny	76.5	71.0	76.4	51.0	**108.9**
Yolov8n	74.8	70.4	75.5	52.7	101.8
Yolov8s	75.6	**71.9**	**76.9**	**53.7**	86.6
RT-DETR	**77.8**	70.3	73.6	51.4	29.9
Yolov10s	74.3	71.4	75.5	51.9	67.3
PD-YOLO	75.4	71.4	76.8	53.6	42.9

The bold values in the table indicate the optimal performance of each method

The mAP@0.5 reached 76.8%, slightly lower than Yolov8s’s 76.9%, but still 3.2% and 1.2% higher than RT-DETR and Yolov10s, respectively. Regarding mAP@0.5:0.95,PD-YOLO also led with a score of 53.6%, outperforming SSD, RT-DETR, and YOLOv10 by 14.1%, 2.2%, and 1.7%, respectively. These results demonstrate that PD-YOLO maintains high detection accuracy even under more stringent evaluation criteria, showcasing the model’s robustness and broad adaptability.

FPS measures the number of image frames processed by the model per second, which is a critical metric for evaluating real-time performance. PD-YOLO achieved FPS values of 42.5 and 42.9 on the CottonWeedDet12 and Lincoln beet test sets, respectively, meeting the requirements for real-time performance. **Table 4** presents the parameter counts and computational complexity of different detection models. Compared to some lightweight models, such as YOLOv7-tiny, which achieved FPS values of 102.3 and 108.9 on the CottonWeedDet12 and Lincoln beet test sets, respectively, with a computational complexity of 13.1 GFLOPs and 6.04M parameters, PD-YOLO has a lower FPS. However, with a computational complexity of 10.6 GFLOPs and 3.96M parameters, PD-YOLO maintains a moderate level of computational complexity and parameter count, achieving a good balance between real-time performance and resource requirements.

**Table 4:** Parameter count and computational complexity of different detection models.

Model	Parameters(M)	GFLOPS(G)
Faster-RCNN	41.41	121.4
SSD	14.50	15.8
Yolov7-tiny	6.04	13.1
Yolov8n	3.01	8.1
Yolov8s	11.20	28.5
RT-DETR	19.89	57.0
Yolov10s	8.04	24.5
PD-YOLO	3.96	10.6

In summary, the PD-YOLO model outperformed other detection models on most metrics in the CottonWeedDet12 and Lincoln Beet datasets, especially in terms of precision and mAP@0.5:0.95. The model provides efficient processing speeds while maintaining moderate computational demands, making it suitable for resource-constrained environments. It achieves an excellent balance between detection accuracy and real-time performance.

### 4.3 Ablation study

We introduced the TIDE metric to comprehensively evaluate the impact and performance of different components on the PD-YOLO model. The TIDE metric allows us to gain a more in-depth and holistic understanding of the role each component plays within the overall detection system, as well as the interpretability of the model design. The effectiveness of weed detection is significantly influenced by the size and quality of the dataset used. Compared to the Lincoln beet dataset, which only annotates two classes (weeds and plants), the CottonWeedDet12 dataset provides detailed annotations of 12 different weed categories, making it more challenging. Given this, we selected the CottonWeedDet12 dataset for the ablation study.

#### 4.3.1 Comparison of different multi-scale feature fusion strategies

Considering the morphological similarity of weeds, we designed the Parallel Focusing Feature Pyramid (PF-FPN). To demonstrate the ability of PF-FPN in multi-scale feature fusion, we conducted comparative experiments with FPN (Lin et al., 2017), PA-FPN (Liu et al., . 2018), BiFPN (Tan et al., 2020), and AFPN (Yang et al., 2023). The experimental results are shown in **Table 5**.

**Table 5:** Performance results of different feature fusion methods.

Model	Parameters (M)	GFLOPS (G)	Precision (%)	Recall (%)	mAP0.5 (%)	mAP0.5-0.95 (%)	FPS
FPN	3.15	9.0	93.4	86.1	93.2	86.8	56.4
BIFPN	2.00	7.1	92.5	87.4	93.2	85.9	97.6
PA-FPN	3.49	9.6	92.4	88.2	94.2	87.7	56.3
AFPN	2.60	8.4	93.7	87.2	93.1	86.8	54.4
PF-FPN	3.96	10.6	94.3	87.0	95.0	88.3	42.5

The experimental results in **Table 5** show that PF-FPN demonstrates significant advantages across multiple metrics compared to other multi-scale feature fusion methods. Specifically, PF-FPN’s mAP@0.5 is 1.8% higher than FPN, 0.7% higher than BiFPN, 1.9% higher than AFPN, and 0.8% higher than PA-FPN. In terms of mAP@0.5:0.95, PF-FPN also achieves the highest score, reaching 88.3%. Compared to other methods, PF-FPN’s mAP@0.5:0.95 is 1.5% higher than FPN and AFPN, 2.4% higher than BiFPN, and 0.6% higher than PA-FPN.

Although the parameter count and computational complexity are higher, resulting in a lower FPS compared to other methods, the significant improvements in precision and recall demonstrate PF-FPN’s superiority in multi-scale feature fusion. These results suggest that PF-FPN can more effectively fuse multi-scale features, leading to a substantial improvement in the model’s detection performance.

**Figure 9:**
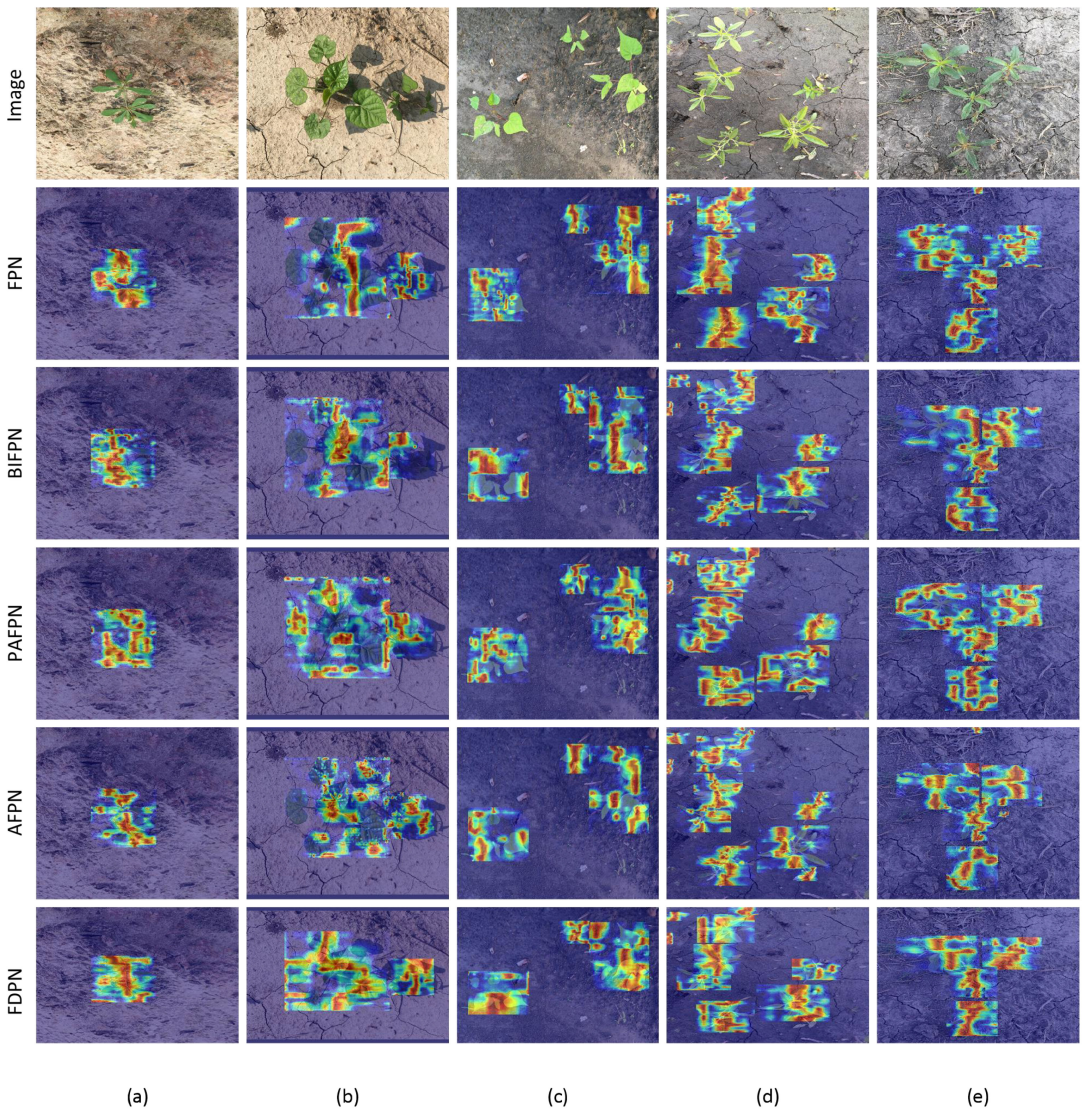
Heatmaps of different images. The first row **(a-d)** shows the original images of different weeds, while the second to fifth rows **(a-d)** display the corresponding heatmaps generated by the model.

We used the Grad-CAM (Selvaraju et al., 2017) heatmap visualization method to present the results in the form of heatmaps, which helps improve the interpretability and reliability of the network. **Figure 9** shows the heatmaps generated by different multi-scale methods. The first row (a-e) represents the original images, while the second to fifth rows show the heatmaps generated by different models. In the heatmaps, darker regions indicate where the model’s attention is more focused. Compared to other methods, the heatmap generated by PD-YOLO shows a more pronounced focus on weed regions. This concentrated attention helps better capture multi-scale features in the image, thereby improving detection accuracy, particularly when dealing with small objects and reducing the likelihood of missed detections.

#### 4.3.2 Comparison of different modules

This section presents the detailed experimental results of the proposed PD-YOLO method. We conducted a comprehensive comparison of PD-YOLO, including the FFAM module, HARFM module, and Dyhead framework, with the baseline model YOLOv8n. We evaluated the performance differences of the baseline model when using and not using FFAM, HARFM, and Dyhead. To investigate the specific impact of each module on model performance, we treated FFAM and Dyhead as independent functional modules based on YOLOv8n, with the FFAM and HARFM modules together forming PF-FPN. Subsequently, by applying the controlled variable method, we analyzed the performance improvements of these modules on the CottonWeedDet12 dataset.

By introducing the TIDE metric (Bolya et al., 2020) for model evaluation and conducting a series of carefully designed ablation experiments, we validated the functionality and performance of each module in PD-YOLO. The TIDE evaluation method identifies the following types of errors in single-class detection problems:

(1) Classification Error (Cls): The model correctly locates the object but misclassifies its category.
(2) Localization Error (Loc): The model correctly identifies the target category, but the bounding box is inaccurate.
(3) Classification and Localization Error (Both): The model makes errors in both classification and localization.
(4) Duplicate Detection Error (Dupl): The model generates multiple high-scoring bounding boxes for the same object.
(5) Background Misclassification (Bkg): The model mistakenly classifies a generated bounding box as the background.
(6) Missed Ground Truth Bounding Box (Miss): The model fails to detect an object that actually exists.
(7) False Positive (FP): The model incorrectly classifies a negative instance as a positive one.
(8) False Negative (FN): The model incorrectly classifies a positive instance as a negative one.

**Table 6** presents the results of ablation experiments conducted on the CottonWeedDet12 dataset, showing significant improvements in several key metrics compared to the original YOLOv8n baseline. Specifically, to address the issue of missed and false detections caused by morphological similarity, the FAFM module was introduced, resulting in increases of 0.5% in mAP@0.5 and 0.9% in mAP@0.5:0.95, demonstrating the effectiveness of FAFM in enhancing small object detection. The HARFM module strengthens weed feature representations, improving the model’s accuracy. The combination of the HARFM and FAFM modules forms PF-FPN, which shows significant improvements in both mAP@0.5 and mAP@0.5:0.95, reaching 94.2% and 87.4%, respectively. This indicates that PF-PFN enhances the performance of multi-scale feature fusion in weed detection. The Dyhead architecture was introduced to improve the model’s stability and accuracy.

**Table 6:** Performance results of the ablation experiments.

Yolov8	FAFM	HARFM	Dyhead	Parameters (M)	GFLOPS (G)	P (%)	R (%)	map0.5 (%)	map0.5-0.95 (%)
✓				3.01	8.1	93.8	87.6	93.3	86.5
✓	✓			3.10	8.4	93.1	89.3	93.8	87.6
✓			✓	3.49	9.6	92.4	88.2	94.2	87.7
✓	✓	✓		3.51	9.3	93.4	87.7	94.2	87.4
✓	✓	✓	✓	3.96	10.6	94.3	87.0	95.0	88.3

"✓" indicates the activation of the corresponding module.

Compared to the baseline model, PD-YOLO showed a 0.6% decrease in recall (R), while precision (P), mAP@0.5, and mAP@0.5:0.95 increased by 0.5%, 1.7%, and 1.8%, respectively. PD-YOLO’s parameter count increased by 0.95M, and GFLOPs increased by 2.5G, resulting in only marginal computational cost increases but significantly better performance across multiple key metrics.

**Figure 10:**
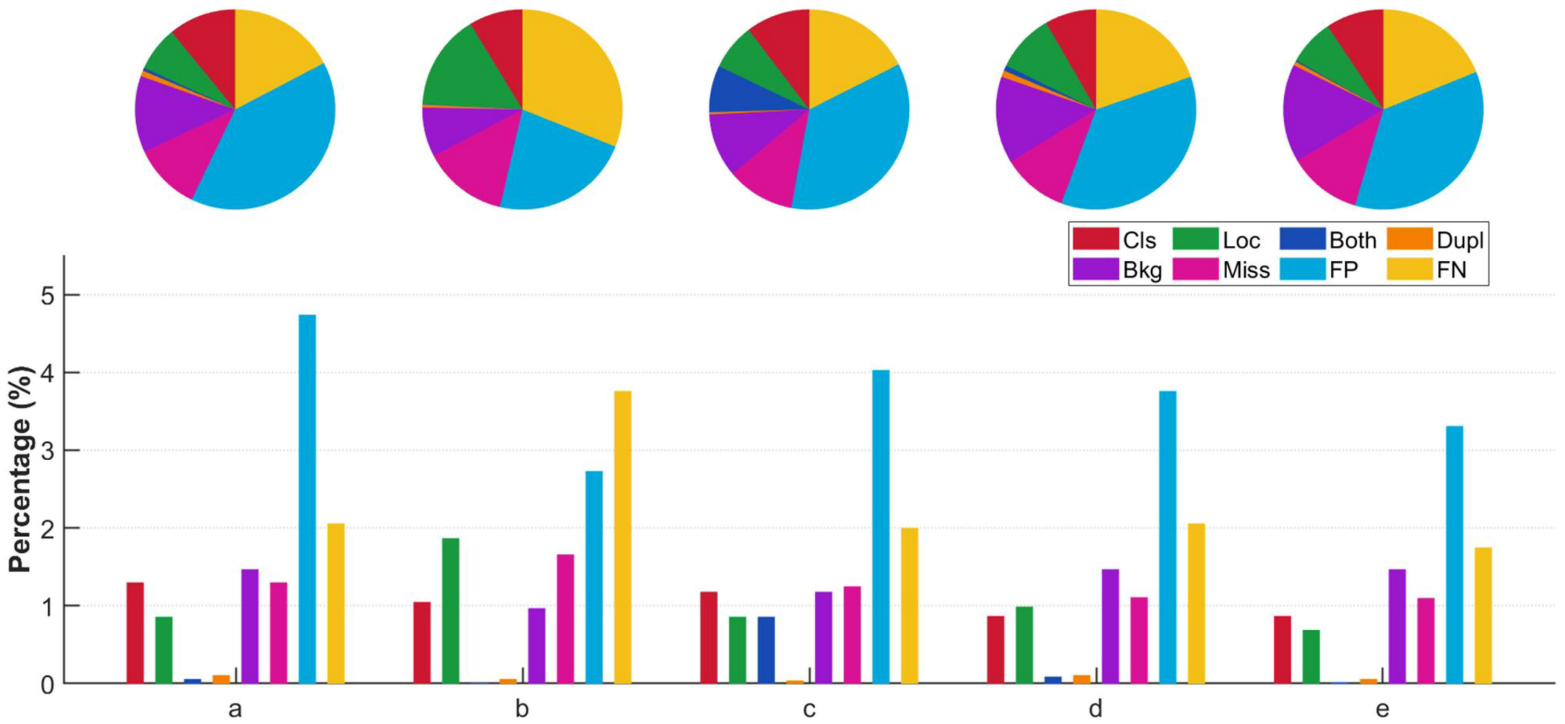
Statistical charts of various TIDE metrics. **(a)** Yolov8, **(b)** Yolov8n+FAFM, **(c)** Yolov8n+Dyhead, **(d)** Yolov8+FAFM+HARFM, **(e)** Yolov8+FAFM+HARFM+Dyhead.

**Table 7** presents the results evaluated using the TIDE method, providing a deeper understanding of the performance improvements in the modified model. The YOLOv8n model had a relatively high background misclassification rate (Bkg). After adding the FAFM module, the Bkg rate decreased by 0.6%. As shown in the TIDE metric statistics in **Figure 10**, the proportion of background misclassification significantly decreased, demonstrating that FAFM effectively reduces background errors in weed detection.

**Table 7:** TIDE metrics of the ablation experiments.

Yolov8	FAFM	HARFM	Dyhead	Cls (%)	Loc (%)	Both (%)	Dupl (%)	Bkg (%)	Miss (%)	FP (%)	FN (%)
✓				1.30	0.86	0.06	0.11	1.47	1.30	4.74	2.06
✓	✓			1.05	1.87	0.01	0.06	0.97	1.66	2.73	3.76
✓			✓	1.18	0.86	0.86	0.04	1.18	1.25	4.03	2.00
✓	✓	✓		0.87	0.99	0.09	0.11	1.47	1.11	3.76	2.06
✓	✓	✓	✓	0.87	0.69	0.02	0.06	1.47	1.10	3.31	1.75

"✓" indicates the activation of the corresponding module.

After combining FAFM and HARFM, the model performed well across multiple metrics, achieving low classification error (Cls) and missed detection (Miss) rates of 0.87% and 0.99% respectively. This indicates that PF-FPN classifies similar weeds more accurately.

The PD-YOLO model excels in multiple aspects, with reductions in Cls, localization error (Loc), both classification and localization errors (Both), duplicate detection (Dupl), missed detections (Miss), false positives (FP), and false negatives (FN) by 0.43%, 0.17%, 0.04%, 0.05%, 0.2%, 1.43%, and 0.31%, respectively, while Bkg remained unchanged. This shows that PF-FPN enhances the accuracy of classifying similar weeds. As shown in **Figure 10**, PD-YOLO demonstrates superior performance in both localization and classification, with only 0.02% of Dupl errors, indicating that the model rarely makes simultaneous errors in classification and localization. This further validates the improvements in the model’s accuracy and its ability to reduce the risks of misclassification, localization errors, and missed detections.

The PD-YOLO model significantly reduces false positive and background error rates while maintaining high precision and recall, thus improving overall detection performance, though its processing speed is slower. These experimental results clearly demonstrate that, compared to the original YOLOv8n algorithm, the PD-YOLO model significantly optimizes and enhances performance, validating the effectiveness of the algorithm improvements proposed in this study.

**Figure 11:**
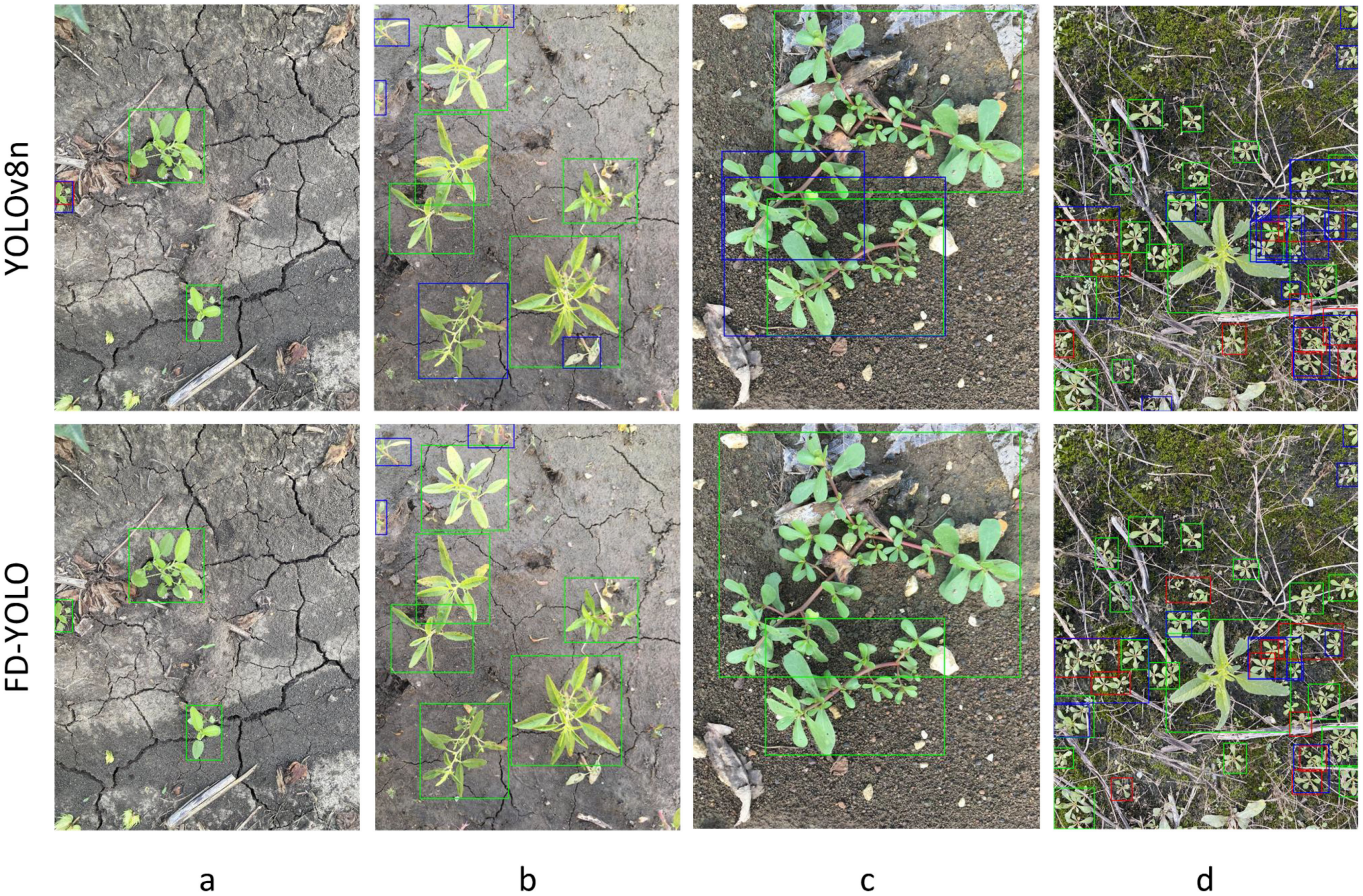
**(a-d)** represent different weed images. The first row shows the YOLOv8 results, and the second row shows the FD-YOLO results. Green boxes represent correct detections, blue boxes represent false detections, and red boxes represent missed detections. Compared to YOLOv8, FD-YOLO reduces missed detections in small target edge weeds **(a)**, morphological differences **(b)**, Mutually occluded weeds **(c)**, and dense occlusion **(d)** scenarios, with more accurate bounding boxes, thanks to the multi-scale feature fusion of PF-FPN and the dynamic attention mechanism of DyHead.

We evaluated the performance of weed detection in various scenarios, including dense weed clusters, partial occlusion, multi-class detection, small-sized weeds, and other complex conditions. The detection results were visualized and compared to observe the algorithm’s ability to identify targets in terms of location, size, and category information. **Figure 11** shows some of the results, where the first row displays the original YOLOv8 results and the second row shows the improved PD-YOLO model’s detection outcomes. In **Figure 11**, due to the small weed target on the left edge, YOLOv8 exhibited missed detections and false positives, while the improved model successfully avoided these issues. In **Figure 11**, the model was able to address detection errors caused by morphological differences within the same weed species, resulting in more accurate bounding boxes. In **Figure 11**, the improved model reduced false positives in scenarios involving occlusion. **Figure 11** presents a challenging sample with dense, small targets, and the improved model significantly enhanced detection performance in this difficult scenario.

With these improvements, the model’s detection performance in complex conditions was significantly enhanced. In practical applications, especially when detecting multiple weed species in complex environments, PD-YOLO can more reliably identify and locate targets, reducing both false positives and missed detections.

## 5 Discussion

This study improves and optimizes the YOLOv8 model, enhancing its performance in weed detection tasks and developing a novel weed detection model, PD-YOLO. The FAFM and HARFM modules were introduced in this study, and based on these modules, a Parallel Focusing Feature Pyramid was proposed to replace the original PA-FPN, improving the model’s feature fusion capability. Additionally, a dynamic detection head was incorporated to enhance the model’s detection stability. These methods improve the classification accuracy of the traditional YOLOv8n model in weed detection and reduce both missed detections and false positives.

Section 4.1 focuses on the performance comparison of different object detection models. The results in **Tables 1** and **2** show that FD-YOLO achieves an mAP@0.5 of 95.0% on the CottonWeedDet12 dataset and 76.8% on the Lincoln Beet dataset. This difference may be attributed to several factors: first, the Lincoln Beet dataset only contains two labeled categories—”weeds” and “beetroot”—while CottonWeedDet12 includes 12 subcategories of weeds, which tests the model’s fine-grained classification ability; second, the weed distribution in the Lincoln Beet dataset is denser, and the target sizes are smaller, leading to increased difficulty in localization in dense occlusion scenarios; furthermore, the images in the Lincoln Beet dataset were captured under the variable field lighting conditions in the UK, with diverse background soil types, further increasing the detection complexity. FD-YOLO’s PF-FPN enhances multi-category feature discrimination through the FFAM and HARFM modules, demonstrating clear advantages on CottonWeedDet12, but the high-density targets in Lincoln Beet require stronger spatial context modeling capabilities. The current model still has room for improvement in the spatial awareness attention mechanism of the Dynamic Head (DyHead). Future work could focus on introducing adaptive resolution adjustment strategies or enhancing spatial attention weight distribution to further improve the model’s performance in high-density scenarios. These differences indicate that FD-YOLO requires fine-tuning or data augmentation for specific environments in cross-regional and multi-crop scenarios.

The results in **Table 4** show that PD-YOLO has fewer parameters and lower computational complexity than lightweight models like YOLOv7-tiny, positioning it as an efficient lightweight model. However, its FPS performance still lags significantly, and it may face challenges such as insufficient frame rates and image blur in high-speed agricultural robots. Further optimization of computational efficiency or the adoption of hardware acceleration solutions is needed. Additionally, future research could explore techniques such as model pruning, quantization, and hardware acceleration to better adapt to low-power embedded devices, ensuring its wide applicability in real-time agricultural applications.

In practical agricultural robotics applications, FD-YOLO can integrate with SLAM technology to enable “detection-navigation-operation” integration. Following Zhang W et al (Zhang et al., 2024), combining 2D LiDAR and visual sensors with YOLOv3 algorithm allows target detection and information mapping onto 2D grid maps for efficient path planning, eliminating computational latency-induced trajectory deviations in traditional models. Researchers applied the trained DIN-LW-YOLO model to autonomous laser weeding robots in strawberry fields, with robot speed set at 0.50 m/s and Intel Realsense D435i camera mounted 600 mm above ground at 30 fps. Field tests demonstrated 92.6% weed control rate and 1.2% seedling damage rate (Zhao et al., 2025). Moreover, FD-YOLO’s lightweight design enables edge device deployment for future weed management. Similar studies deployed customized YOLOv7 models on NVIDIA Jetson Xavier NX platforms, integrating robotic frame spraying systems that recognize Amaranthus palmeri in cornfields for real-time spot spraying (Balabantaray et al., 2024).

Furthermore, the ablation experiments validated the roles and necessity of each module and their impact on model size, as discussed in Section 4.2. The experimental results demonstrated the impact of each module on the model’s recognition accuracy, as well as a comparison of different feature pyramids. In PF-FPN, the excessive upsampling and downsampling during feature aggregation and distribution by the FFAM module led to a loss of detailed features. Therefore, the introduction of the HARFM module reduced this loss, showcasing efficient feature fusion capabilities. Despite the increase in computational cost and detection speed, the improvements in multi-scale feature fusion and small object detection enable the PD-YOLO model to perform well in scenarios involving high-density, partial occlusion, and multi-class weeds. However, in practical applications, environmental adaptability still needs to be considered. The robustness of the model under low-light conditions or extreme weather has not been validated. The current dataset is primarily based on natural lighting conditions, which may limit the model’s stability in complex lighting scenarios.

The systematic analysis of the TIDE metrics provides clear directions for model optimization. Experimental results show that FD-YOLO significantly outperforms the baseline model in terms of classification errors and localization errors, but there is still room for further optimization. To address classification errors, future work could introduce fine-grained feature alignment strategies to enhance the model’s ability to distinguish between morphologically similar weeds. The residual localization errors may stem from blurred object boundaries in complex occlusion scenarios, which could be improved by integrating deformable convolutions or refining the bounding box regression loss function to boost localization accuracy. Additionally, the background misdetection rate remains relatively high, indicating that the current model lacks sufficient suppression of background noise such as soil texture. To address this, more diverse background samples should be included during data augmentation, or a lightweight background-aware attention module could be designed.

## 6 Conclusions and future work

This study proposes PD-YOLO, a novel computer vision method specifically designed for real-time weed detection. The architecture of PD-YOLO combines the Parallel Focusing Feature Pyramid (PF-FPN) and Dyhead framework, built upon the YOLOv8n framework. The FAFM module optimizes feature fusion, enhancing the model’s representational capabilities, while the HARFM module strengthens weed-specific features, improving weed identification. The PF-FPN network, developed with consideration of weed morphological characteristics, serves as an effective feature fusion network for weed detection. The Dyhead framework improves the design of the detection head, ensuring both accuracy and stability in detection results.

The research results show that, compared to the baseline model, PD-YOLO improves mAP by 1.7% and 1.8% (at thresholds of 0.5 and 0.5-0.95, respectively). While maintaining a lightweight structure, PD-YOLO outperforms current mainstream object detection algorithms, demonstrating superior performance. Moreover, although the model’s detection speed meets real-time detection requirements, there is potential for further optimization in real-world field environments.

Future research will focus on the following directions:

(1)Data Augmentation and Multimodal Fusion: Integrating multispectral imaging data to enhance the model’s detection capability under complex lighting and occlusion conditions, and expanding data diversity through synthetic data augmentation (e.g., simulating rain, fog, and shadows).

Lightweight and Efficiency Optimization: Developing an FD-YOLO-Tiny variant that combines the vMamba (Zhu et al., 2024) architecture to improve the model’s backbone, reducing computational overhead while maintaining accuracy, and adapting it for deployment on edge devices.

(2)Error-Driven Model Improvement: Based on the analysis results from the TIDE metrics, specifically optimizing the false negative and false positive modules. This can be achieved by strengthening the training of negative samples, improving the loss function, or adjusting the post-processing stage of the detection algorithm to reduce misdetections and missed detections.

(3)Cross-Scene Validation and Transfer Learning: Expanding experimental validation to include different crops, such as corn and wheat, and varying agricultural environments. Combining transfer learning techniques to enhance the model’s generalization ability, ensuring its practicality in diverse agricultural settings.

## Data Availability

Publicly available datasets were analyzed in this study. This data can be found here: (1) CottonWeedDet12, Dang F, Chen D, Lu Y, et al. YOLOWeeds: A novel benchmark of YOLO object detectors for multi-class weed detection in cotton production systems. Computers and Electronics in Agriculture, 2023, 205: 107655.(2) Lincoln beet, Salazar-Gomez A, Darbyshire M, Gao J, et al. Towards practical object detection for weed spraying in precision agriculture. arxiv preprint arxiv:2109.11048, 2021.
